# Charles Gardiner Guthrie

**Published:** 1860-01

**Authors:** 


CHARLES GARDINER GUTHRIE died at London during the early part of last autumn, at the age of forty-two. He was a son of the late Mr. James Guthrie, the celebrated army surgeon, and had long held the post of lecturer on surgery at the Westminster Hospital. He was the author of several learned papers on ophthalmic medicine and surgery, and of an able treatise on cataract.

